# Utilization of health-related data in the regional context for health service planning in the Federal State of Brandenburg, Germany—a qualitative study

**DOI:** 10.1007/s43999-024-00050-0

**Published:** 2024-09-25

**Authors:** Charlotte M. Kugler, Daniela Koller, Felix Muehlensiepen, Alexander Pachanov, Anna Kuehne, Dawid Pieper

**Affiliations:** 1 Institute for Health Services and Health System Research, Faculty of Health Sciences Brandenburg, Brandenburg Medical School (Theodor Fontane), Rüdersdorf, Germany; 2 Center for Health Services Research, Brandenburg Medical School (Theodor Fontane), Rüdersdorf, Germany; 3 Institute for Medical Information Processing, Biometry, and Epidemiology (IBE), Ludwig-Maximilians-Universität München, Munich, Germany; 4 Chair of Public Health, Centre for Evidence-Based Healthcare, University Hospital an Medical Faculty, TUD Dresden University of Technology, Fetscherstr. 74, Dresden, 01307 Germany

**Keywords:** Regional health planning, Health atlas, Unwarranted variation, Geographic variation, Qualitative study, Data sources

## Abstract

**Background:**

Utilizing regional health data goes hand in hand with challenges: can they be used for health planning, are they applicable to the relevant topics? The study explores current data utilization and needs of stakeholders working in regional health services planning.

**Methods:**

We conducted 16 semi-structured expert-interviews with stakeholders of regional health planning in Brandenburg, a federal state in the north-east of Germany, by telephone or online-meeting tools between 05/2022 and 03/2023. The data were analysed according to qualitative content analysis.

**Results:**

Utilization of data sources depends on individual knowledge and personnel resources instead of being guided by standardized procedures. Interviewees primarily use internal data; some use many different platforms, studies and reports. Regional health-related data are used for reliable health planning, to prepare resolutions, draft contracts, but also for events and requests from policy makers or the press. Challenges exist in terms of availability, awareness, and acceptance of the data, perceived applicability, the ability to use it and the utilization itself. Many regional health planners indicated they would appreciate a regional integrated cross-organizational data source if the benefits for health planning outweighed the efforts.

**Discussion:**

Actors in health planning primarily utilized their own data for planning; additional data sources are not available or the level of aggregation is too high, not known by them or are often not used due to a lack of time. A standardized regional monitoring would require the definition of indicators as well as the strengthening of cross-sectoral planning.

**Supplementary Information:**

The online version contains supplementary material available at 10.1007/s43999-024-00050-0.

## Background

A spatial view on health and disease has a long tradition in Germany and some other countries, starting with the distribution of epidemics and becoming very popular during the COVID-19 pandemic [1–3]. Health-related data from diverse primary data sources, such as epidemiological outcomes and health service utilization, can be analysed for specific spatial areas (e.g. counties, federal states) and compared to other areas. These analyses reveal regional variations in health outcomes and services [1, 4–6]. Unwarranted healthcare variation refers to “patient care that differs in ways that are not a direct and proportionate response to available evidence; or to the healthcare needs and informed choices of patients” [7]. There are multiple ways how the variation can be presented, thematic maps are often chosen as an informative way for descriptive presentation of variation. A popular tool to show regional variation are so-called ‘health atlases’ which differ in analytical dimensions, levels of aggregation, and target groups [7, 8]. Yet, there is no consensus about the definition of health atlases [1, 8]. Prominent health atlases are The Dartmouth Atlas of Health Care [9, 10] and the Health Atlas of the German Central Research Institute for Ambulatory Health Care [11].

While there are numerous regional health-related data sources available, it remains unclear how these support health planning and what challenges exist. One major challenge internationally known is the limited availability of data on a small-area scale, either because regional information is simply not available or due to data protection issues. Another issue might arise to the so called modifiable aria unit problem (MAUP), summarizing potential difficulties to interpret data because of different zoning or different levels of aggregation, and potential incompatibility of spatial units [2, 12].

In Germany, various health atlases use county or federal state level aggregation focusing on health services, epidemiology, structure specific subjects, mortality, or sociodemographic characteristics [8]. Brandenburg is a predominantly rural federal state in the north-east of Germany with urban areas near the capital Berlin [13, 14]. From herein, we will refer to the Federal State of Brandenburg as ‘Brandenburg’. In Brandenburg, health-related regional data also exist, such as the Health Platform Brandenburg, addressing health topics of children, school enrolment, adolescents, inpatient care at the county level [15]. To date Brandenburg lacks a comprehensive platform or health atlas including diverse data on health outcomes and services similar to those found in other federal states like Bavaria [2, 16]. Even when data on variation are available, questions arise about their utilization. While visualization of data through health atlases initiates a discussion on unwarranted variation [17], it is crucial to move from information to action. A framework by Schang and colleagues [18] empathises prerequisites for research use in health planning, including awareness, acceptance, perceived applicability, ability to use. Only when these are met, health atlases information can be used to identify unwarranted variation, assign responsibility for action, and making decisions to decrease variation. Challenges like limited applicability – for example due to unclear interpretations of single indicators for the entire pathway – and difficulties in discriminating between warranted and unwarranted variation often hinder the use of health atlases in health planning [18].

### Objective

The objectives of this study were to identify 1) data sources currently utilized by different stakeholders for health planning in Brandenburg, 2) current challenges with utilization of these data, and 3) to explore unmet needs of stakeholders of regional health services planning. Furthermore, this study aims to provide insights whether a comprehensive health atlas or a cross-organizational database is needed in Brandenburg.

## Methods

We registered a protocol in the Center of Open Science (OSF) registry (https://osf.io/mya6r). We followed O'Brien et al.’s *Standards for Reporting Qualitative Research* [19] when reporting our study (Appendix 1).

We conducted individual semi-structured interviews with stakeholders of regional health planning in Brandenburg interpreted by content analysis [20].

### Researcher characteristics and reflexivity

The project team included health service and public health researchers and a health geography scientist. Therefore, the study was carried out by neutral, external scientists, who were mostly new to the context of Brandenburg. The research question emerged by studying health care reporting of Brandenburg and comparing it to other federal states of Germany. We noticed that Brandenburg has no comprehensive health atlas. At the same time, it was unclear how existing health atlases in Germany are used for health planning and which data are actually needed.

### Sampling strategy

The German health system is characterized by its fragmented medical care across different sectors [21]. Specifically, planning and budgeting for hospital and outpatient care operate independently, lacking integration with other services like health promotion, prevention, rehabilitation etc. While the federal level sets the overall legal framework, state governments are responsible for hospital planning and public health services. Usually, there is one local public health authority per county, whose responsibilities include planning of health promotion or prevention interventions, infection control and health monitoring. Regional Associations of Statutory Heath Insurance Physicians are responsible for planning outpatient physicians and psychotherapists [21]. We addressed different stakeholders of regional health planning in Brandenburg (Table 1).

**Table 1:** Contacted organizations of regional health planning, ^a^ contacted but did not respond

1) Federal State Committee according to paragraph 90a Social Code Book five (short: 90a committee) which was built to answer questions regarding cross-sectional health services derive appropriate recommendations in Brandenburg. It includes
a. Ministry of Social Affairs, Health, Integration and Consumer Protection of the Federal State of Brandenburg
b. Association of Statutory Health Insurance Physicians
c. Federal State Associations of Health Insurances
d. Brandenburg State Hospital Association^a^
e. Representatives of the Central Municipal Associations in Brandenburg^a^
f. Organizations from health professionals: Brandenburg’s Medical Council, the Eastern German chamber of Psychotherapists, the North-East German Nurses Association^a^
g. Brandenburg’s Council for handicapped people^a^
h. Federal Association of Private Providers of Social Services
2) Local public health authorities (18 in total)
3) The Health Objectives Unit carried by the Berlin-Brandenburg Association for Health

Organizations were contacted by e-mail (individually or through mailing list) and postal service simultaneously; a reminder was sent by e-mail after approximately four months. Organizations themselves selected the most suitable person to participate. Because interviewees have useful networks, we also applied snowball sampling. We ended recruitment once 1) data saturation was reached and 2) participants of all three target groups were included [20]. 

### Interview guide

A semi-structured interview guide was developed within the team based on five key topics (Table 2). The full interview guide is available in Appendix 2. Before the interview date, we sent key questions to the interviewees to allow preparation of the interview. The two first interviews served for piloting but there was no need to adjust questions.

**Table 2:** Interview guide topics

Topic number	Key question
1	Please describe what comes to your mind when you hear the term "health atlas" Could you please describe your experience with health data with a regional context? (e.g. health atlases, databases, table volumes, reports, queries to data holders)
2	If you were to make an assumption: For which aspects of health care does variation exist in Brandenburg?
3	Please describe how your organization uses data sources during health planning
4	Please describe the challenges you face in healthcare planning with regard to data sources
5	What do you think of a cross-organizational data source for regional health planning?

### Stakeholder involvement

The project idea, research questions and interview guide were discussed with organizations responsible for health planning or health monitoring in Brandenburg and other federal states in Germany to gain their perspective and input. There were no amendments.

### Data collection

The interviews were conducted by telephone or online-meeting tools (Cisco Webex) depending on the preference of the interviewees (CMK) between May 2022 and March 2023. All interviews were recorded (Online Meetings via Cisco Webex, San José, USA; telephone interviews via Olympus Digital Voice Recorder VN-541PC, Shinjuku, Tokyo, Japan).

### Participants

We conducted 16 expert interviews that lasted on average 51 min (range: 29 to 65). Experts were primarily employees of organizations within the cross sectional federal state committees (seven interviewees working at six different organizations; mostly in management positions) and of local public health authorities (seven interviewees at seven different local public health authorities; mostly health coordinator, psychiatry coordinator, medical officer). Table 3 gives an overview of interviewed organizations.

**Table 3:** interviewed organizations

Type of organization	Number of interviews
Cross sectional Federal State Committee	7
Local public health authority	7
Health Objectives Unit	1
Working group for outpatient counselling and treatment centres	1
**Total**	**16**

### Data processing and analysis

Recordings were transcribed verbatim by a professional company (abtipper.de, scryvo.com). After accuracy checking of transcriptions (AP), audio recordings were deleted. MAXQDA software (Standard Version 18.2.4, Verbi GmbH) was used to analyse and code the data. First, all transcripts were read to gain an overview of emerging patterns in the data. Qualitative content analysis was used to aggregate and order the main outcomes of the interviews [20]. Deductive categories of the coding tree were developed based on literature on utilization of health atlases [18], health atlases [8] and topics of the interview guide. (Sub-)categories emerging from the data were added inductively. The coding tree was tested on two randomly selected transcripts by two authors (CK, AP), and adjustments were made following discussions. Two further randomly selected transcripts were coded independently, results were compared during discussion and the final coding tree was developed. Given the acceptable interrater reliability (kappa of ≥ 0.8) between two authors, subsequent interviews were individually coded by one author (CMK), with any uncertainties being addressed through discussion. After coding of all interviews, the categories were discussed within the full team. Main quotes were selected for publication and translated to English (CMK, AP, Appendix 3).

### Ethical considerations

The study has received an ethics waiver by Brandenburg Medical School, Germany (Medizinische Hochschule Brandenburg, waiver no. E-01–20220303). All participants confirmed their written informed consent for participation.

## Results

### Utilization of health-related data sources

Utilization of data sources depended on individual knowledge and personnel resources. The interviewees primarily used their own (internal) data, for example utilization of own services, available surveys of the paediatric and adolescent health service or socioeconomic data.

Own data collections included surveying of organizations or stakeholders, survey of citizens, pupils or association members for their needs and experiences as well as data collections for health reporting. Some interviewees utilized many different platforms, studies and reports that differed in terms of content and providers (Table 4). Environmental data have hardly been used for health planning to date, but, according to interviewees, they are becoming more relevant. Exceptions of utilizing environmental data include integrative local planning or the quality of drinking water and bathing lakes for which local public health authorities are responsible for quality assurance.

**Table 4:** Mentioned data utilized for health planning

Contents	Providers	Dashboards	Studies / Reports
• Population statistics • Cause of death statistics • Commuter flows • Nursing homes • Vaccination rates • Infectious diseases • Population forecasts • Shared accommodations (for refugees) • Frequency of diseases • Quality indicators • Professionals • Socio-demographics • Accessibility • Emergency care • Birth cohort (insurance-related data basis containing approx. 12% of all statutory-health-insured persons) • Waiting times • People with disabilities • Crime statistics • Addiction aid statistics	• State and federal statistical offices • Health insurance funds • Brandenburg Association of statutory health insurance physicians or dentists • Hospital diagnosis statistics • Robert Koch Institute (a federal government agency responsible for disease control and prevention) • Scientific institute of the general local health insurance Funds (WIdO) • Federal institute for Research on Building, Urban Affairs and Spatial Development (BBSR) • Hospital Association • Central Institute of the Association of statutory health insurance physicians • IGES institute (a private institute with research on health, mobility, education and living) • Registry offices • Federal offices (e.g. Brandenburg’s Office for social affairs and care) • Bertelsmann foundation (research on health and education) • The Generation and Educational Science Institute (Genesis) • Accident insurances • Medical statistics of the medical association or dental association • Pension insurances • Municipal hospitals	• Brandenburg health platform • SAHRA Platform (smart analysis health research access) • The Information System of the Federal Health Monitoring • Gapminder.org • COVID-19 Dashboard	• Study on young people and substance use in Brandenburg • Brandenburg social indicators • COPSY (investigates the impact of the COVID-19 pandemic on the mental health of children and adolescents in Germany) • State hospital plan • AdiRaum (development of small-scale meaningful data from school entry examinations for nationwide health monitoring using the example of overweight and obesity prevalence) • EMOTIKON (primary school sports, annually recordings of motor fitness of third-year pupils) • HBSC study (Health Behaviour in School-aged Children) • GEDA survey (current health in Germany) • Commissioned expertises • Cross-federal-state health report provided by an insurance • KIGGS study (on the health of children and adolescents in Germany) • BELLA study (survey on well-being and behaviour of children and adolescents) • Alcohol atlas • Addiction support report for Brandenburg

Regional health related data were primarily utilized for reliable health planning including public health interventions and health services.

*„We are currently using, specifically for Project …, to more effectively control our interventions. So, there has always been a tendency, (…) [towards] a scattergun approach. (…) And the evaluation of our data analysis has shown that this is a mistake. So, we have much greater affection, meaning worse health data, in areas farther away from Berlin.“ (T06 – local public health authority)*

Regional health-related data were also utilized for presentations at public events and in committees for example to inform qualified personal, or to prepare resolutions at regional health conference or government. The data were also utilized for public relations and lobbying, for designing contracts (for example an agreement for rheumatologists, payment negotiations) and to answer enquiries from decision-makers or within internal research projects.

*„When presenting the Mobility Atlas. (...) This was also presented to the press. And so we use, yes, we publish in various areas, but we also use it for discussions, also for position papers and also as part of events.” (T15 – member of 90a committee)*

Interviewees described regional health-related data helped them to evaluate the population’s health and whether health services were appropriate or to develop a strategic plan. Regional health-related data were also utilized for funding applications, for forecasts and processing of complaints. Nursing homes used is to proof availability of medical care for their residents.

### Current challenges with utilization of the data and unmet needs of stakeholders of regional health planning

**Fig. 1:**
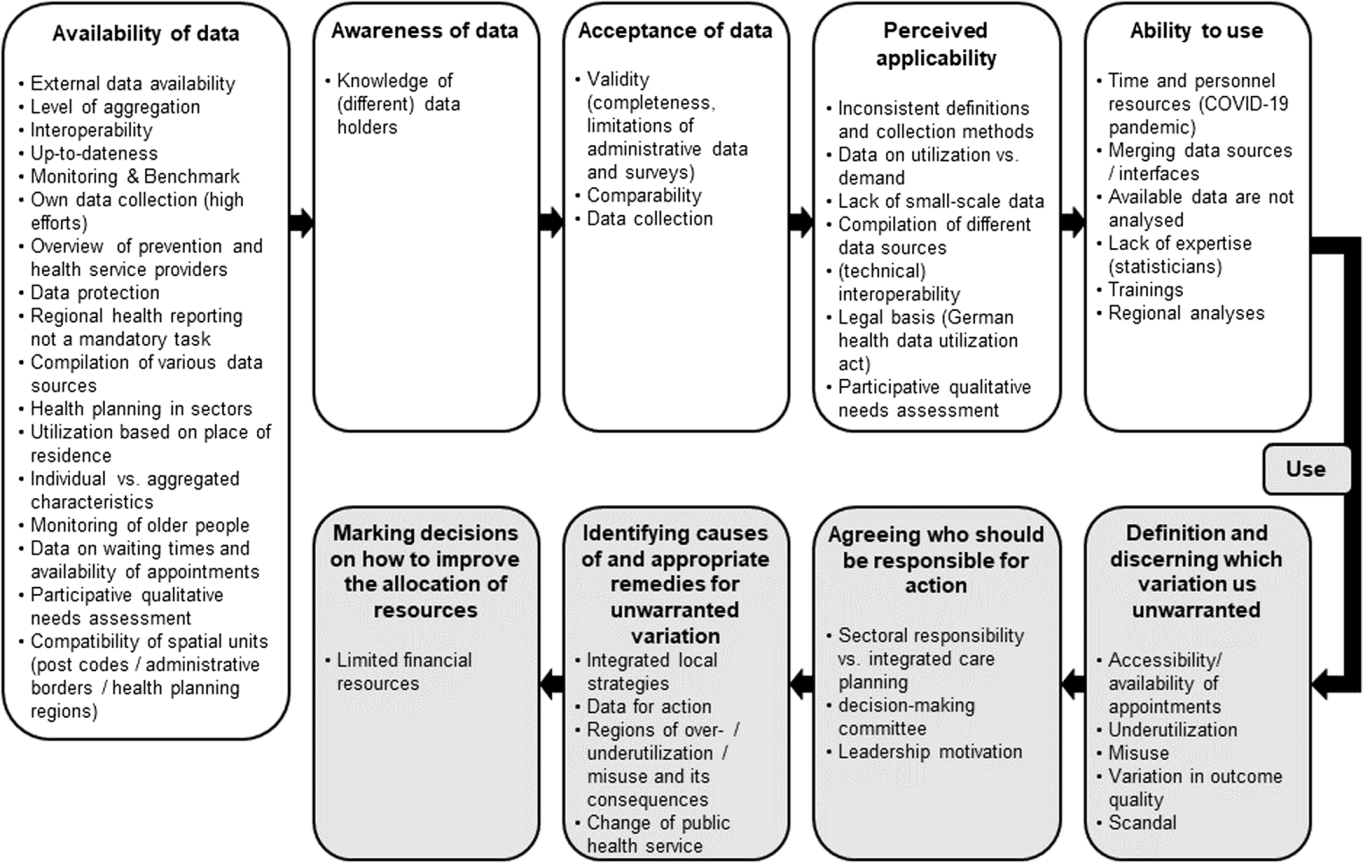
Challenges for utilization of regional health-related data

Interviewees reported challenges for utilization of regional health-related data that corresponded to all categories within the framework for moving from data on geographic variations to resource allocation decisions [18] to which we added the category “availability” (Fig. 1). Below, we will report challenges following this framework.

#### Availability of data

Regional health-related data were often not available, for example, interviewees had no access to external data, the level of aggregation was too high (granular data were not available) or data were out of date. Interviewees reported missing interoperability between different data sources and missing compatibility of different spatial units that hinder utilization across organizations or combination of different data sources.

Many organizations maintain their own lists of regional medical service and care providers, because an overview of (intersectoral) medical service, care and prevention providers is lacking.

*„The structures of statutory health insurance providers are sort of black box for us. (...) We don't know where, which health insurance is offering a health promotion program.“ (T06 – local public health authority)*

A frequently reported unmet need was to benchmark one's own region with other regions across different indicators and to have a regular health monitoring not only for children.

#### Awareness of data

Interviewees reported it was challenging to know (many) different data holders and where they could find regional health-related data.

*“I think we have a lot of data. I don't think everyone is aware of the data [sources].” (T16 - Member of 90a Committee)*

#### Acceptance of data

Challenges existed in terms of validity. For example, an interviewee reported availably survey data of children and adolescent health were only incompletely entered into the federal database until the deadline for federal analysis thereby limiting comparability and validity. Interviewees mentioned general limitations of surveys such as the recall bias, human mistakes during data collection and entry, and limitations of administrative insurance data such as generalizability. Interviewees also reported limited comparability because definitions of indicators may change or they were not equal in different regions.

*„I've also noticed that it's sometimes not so easy to really determine developments and development trends for the period that you would perhaps be interested in, simply because definitions for key figures and indicators have changed and then, quite simply, comparability is no longer given. This happens relatively often. In part, it is also understandable, in part, you set the definition yourself, rewrite it, simply because it is no longer up to date and no longer corresponds to the medical state of the art or the public health state of the art.” (T12 – Member of 90a Committee)*

#### Perceived applicability

Interviewees described limited applicability of regional health-related data, because of inconsistent definitions and collection methods and lack of small-scale data. According to participants, often, only data on utilization are available, but this does not correspond 1:1 with needs; e.g. no data are available on people with needs who are not receiving care within the healthcare system. Compilation of different data sources was perceived as complex and time-consuming especially due to lacking (technical) interoperability (available data are not machine-readable). In addition, interviewees mentioned a lacking legal basis to combine data held by different organizations but hoped for the German health data utilization act which was prepared at the time of the interviews.

*„So the districts are too coarse for us. (...) Brandenburg was cut in such a way that many Brandenburg districts still have a slice of the Berlin metropolitan area, right. (laughs) And then there's the periphery, and that's, um, very difficult now, of course. It is of course to be expected that we have a strong heterogeneity within such a district.” (T16 – Member of 90a Committee)*

#### Ability to use

Especially local public health authorities reported time constrains and limited personnel resources as challenging for using health-related data for health planning. Several interviewees described that, especially during the Covid-19 pandemic, when staff was deployed to other functions, available regional health-related data were not analysed. In addition, merging data sources was challenging, and interviewees reported there was a lack of expertise (e.g. no statisticians) which is why it was suggested to train personnel in collecting, analysing and utilizing regional health-related data (qualitative and quantitative).

*„The evaluation of this data and we have an enormous amount of it at the local public health authority. The software we work with could be used to record and evaluate it statistically, including for regionality. But we simply don't have the time to deal with it.“ (T03 – local public health authority)*

#### Use

Interviewees had difficulties in defining unwarranted variation. They would focus on accessibility or availability of appointments at health services if these data were available. The identification and definition of underuse and misuse of health services was not perceived as straightforward, because mathematical approaches do not cover the whole picture. Some interviewees focused on variation in outcome quality or public ‘scandals’ on which was reported in the news for defining unwarranted variation.

*“So these are always purely numerical figures, and they can be somewhat difficult to assess - I would say. Just because there is a service doesn't mean they will actually get there, get an appointment, or receive the care they need. (…) but nobody has a real overview.” (T13 - Member of 90a Committee)*

According to interviewees, the sectoral responsibility hindered agreeing on who should be responsible for action and they aspired integrated (care) planning. In addition, high motivation of leadership was perceived as key for pushing actions.

*„Let's put it this way, integrated care or integrated planning in city administration is unfortunately somewhat challenging.“ (T05 – local public health authority)*

Interviewees described that integrated local strategies would help identifying causes of and appropriate remedies for unwarranted variation. One person thought that in Germany, there is a need for reorganization of the public health service to move from “data for action”. The identification of regions with over- and underutilization of health services and their consequences on health were perceived as challenging.

Finally, interviewees saw limited financial resources as a barrier to make decisions on how to improve the allocation of resources.

### Insights of the need of a regional comprehensive health atlas or a cross-organizational data source

Some interviewees defined health atlases as collection of statistical health-related indicators (including social determinants of health) allocated to spatial units allowing comparisons and evaluations, but others thought of a cartographic overview of medical service and care providers including their specialization.

*„For me, it is basically a collection of statistical data, key figures and indicators that I can use for my work and, in particular, for my planning. Above all, of course, not only in relation to the entire federal state of Brandenburg, but of course for our local public health authority. And, in the best case, also for the local neighbourhood.“ (T04 – local public health authority)*

Most interviewees had a positive view on the idea of a cross-organizational data source, which would combine available regional health-related data across different organizations (i.e. local public health authorities, insurances, association of statutory health insurance physicians, nursing homes, hospitals etc.). They intended to utilize such a cross-organizational data source for reliable integrated regional health and care planning, to confirm feelings about variations with data, for public relations and for better comparison of own’s regions (e.g. district) to others due to more transparency.

*„In my opinion, that would be the ultimate for us, to really look at a small scale, in the community XY, age structure, incidence of illness, care needs, nursing diagnoses, that would be something where you say, according to the data, the topic is to be focused on in the community for corresponding projects. (...) That you really use the resources, the funds, that are available somewhere, sensibly.” (T08 – local public health authority)*

Other interviewees were undecided or had a rather negative view on the idea of a cross-organizational data source because they saw challenges regarding data protection, a legal basis and high efforts needed for such a project because of diversity of data holders that would need to agree on common goals.

*„If you really have a common objective by evaluating this data, then I think that's good. But we don't have that, at least not at the moment.“ (T01 – member of 90a committee)*

Interviewees emphasized the need to standardize definitions of indicators across areas of responsibility, to ensure interoperability and to provide appropriate interfaces to work with the data. Among the interviewees, there were uncertainties about data quality, updating and preparation of data, funding and the relation to the health data utilization act or the European health data space. Interviewees expressed requirements for the design of a cross-organizational data source (e.g. type of analysis, level of aggregation, indicators, updating, download and alarm functions), which however would need to be concretized. According to participants, the Ministry or Federal State Office of Health should be responsible for realization. Requirements for realization included political will and funding.

In sum, interviewees favoured a regional cross-organizational data source if the benefits for health planning outweighed the efforts.

## Discussion

### Main results

The study indicates that the awareness and utilization of data sources for health planning in Brandenburg, Germany, are very much dependent on individual knowledge and personnel resources. Besides for health planning, regional health-related data are also utilized for preparation of resolutions and public relations. A wide range of challenges is associated along the framework for moving from data on geographic variations to resource allocation decisions [18].

### Strength and limitations

The study has some limitations. First, there are limitations inherent to qualitative research such as limited comparability of answers and restricted generalizability, especially since we explored the needs in Brandenburg influenced by specifics of the German health care system and regional factors. The credibility was further supported by asking multiple and follow-up questions regarding each topic and by encouraging participants to provide examples. However, participants did not have the possibility to check the final manuscript for correctness of interpretation. Despite the possibility of a selection bias as persons may have been more inclined to participate if already familiar with utilizing health-related regional data, there was a heterogeneity among the interviewees in their experience of utilizing health-related data for health planning. This led to reaching data saturation, although the Brandenburg State Hospital Association did not take part in the study and the number of interviews was smaller than in other studies [18]. One of the strengths of the study is that organizations responsible for health planning or health monitoring were involved before starting the interviews.

### Further needs

#### Cartographic overview of medical and care providers

A primary challenge identified is the lack of a comprehensive overview of medical service and care providers, similar to a “health version of Google maps”. The Austrian Health Information System (ÖGIS) comprises information on the health system (health, care and prevention providers including their specialization, their utilization and quality), though it is not publicly accessible and analyses are subject to charges [22, 23]. Due to the sectoral German health system (outpatient, inpatient acute care, nursing facilities, rehabilitation providers and public health services) and divergent responsibilities, there is currently no such overview. Therefore, health planning organizations maintain their own lists of regional medical service and care providers resulting in unnecessary duplication of work. A publicly accessible nationwide overview could help health planners but also inform patients, although opinions differed among the interviewees as to whether the data should be publicly accessible.

#### Regional health-related data

Many interviewees primarily utilized internal data sources available through their own organization. In a study investigating the use of the NHS Atlas of Variation, there was also a preference to work with internal raw data (in contrast to using the atlas) due to up-to-dateness and more details [18]. The sectoral health system in Germany leads to a wide range of data holders [24]. Integrated health planning necessitates incorporating data from other policy areas and requires cooperation among different policy departments to formulate actionable objectives [25]. Other countries have successfully identified relevant indicators: in Switzerland, for example, a multistep procedure was employed to designate relevant indicators [26]. In Italy, a voluntary based governance tool allows benchmarking for about 400 indicators selected based on their potential to inform managers and policymakers [27]. In Finland, a web-service provides benchmarking for 455 indicators [28]. In Germany, an indicator set on the federal state level exists since 2003 [29] and currently, the RESILIENT project in Dresden aims to develop a small-scale integrated health index [30]. Interviewees discussed a regional cross-organizational integrated database distinct from a health atlas allowing more flexibility which is exemplified by countries as Austria [23] and Brazil. The latter contains 146 national public health and healthcare databases with open and unprocessed data from different sources and formats [31].

Interoperability of different data sources emerges as a crucial technical challenge. In Switzerland, the program “DigiSanté” aims to reach interoperability between treatment, reimbursement, research, and administrative health care data, which are to date stored in unconnected, inconsistent data silos by defining standards and providing infrastructure [26, 32]. In Germany, initiatives like the Health Data Lab provided by the Federal Institute for Drugs and Medical Devices (BfArM) are planned to provide researchers with access to extensive health insurance data. Still, achieving interoperability remains a significant challenge because there is no Unique Personal Identifier (UPI) allowing combination of (pseudonymized) data on the same person across different databases [26, 33, 34].

Interviewees, particularly those at district or municipal public health authorities, complained on insufficient granularity of available data. Data collection at the district level is often limited or non-existent [2]. In 2018, an expert report proposed a small-scale monitoring system for planning outpatient physicians in Germany that allows a comparative regional assessment of relevant indicators [35]. This system should also incorporate indicators for other health sectors (e.g. health promotion, inpatient services) to allow integrated health planning. Italy, Austria and Spain demonstrate successful models of interregional data sharing and benchmarking at several levels of spatial resolution, facilitating comprehensive performance evaluation of health systems [22, 27, 36].

Visualization plays a pivotal role in communicating with decision makers [18, 27]. Dartboards allow easy benchmarking across multiple indicators [35]. Italy uses both, classical (histogram, map) and innovative (dartboard) visualizations for or regional performance assessment. Recently, it incorporated a new tool called the "stave" to represent care pathways' performance emphasizing the contribution of each organization to overall pathway results [27].

From data to action, leadership and shared goals are paramount in driving awareness and utilization of health-related data [18]. In countries like Brazil and Spain, tailored reports are provided to decision makers [31, 36]. A solution may also be to use hospitals or teams as the unit of analysis and to identify evidence-based strategies for change [37].

Responsibility for health planning between national, federal, municipal and committees of self-administration raises question about appropriate roles for each. To achieve integrated regional planning, cross-sectoral planning structures (e.g. within the 90a committee or regional health conferences) should be promoted. Essential prerequisites for effective health planning include allocating adequate resources and mandates within the structure [38].

## Conclusion

Many actors in health planning primarily utilize internal data sources; external data sources are either not available or not granular enough, unknown to them, or not used due to lack of time. For a regional, cross-organizational data source or regional monitoring, common goals and indicators would first need to be defined, and interoperability ensured. This, along with strengthening cross-sectoral planning, would be a possible way to achieve integrated regional health planning in the future.

## Supplementary Information

Additional file 1: Appendix 1. Standards for Reporting Qualitative Research. Appendix 2. Interview guide. Appendix 3. Translation of selected quotes.

## Data Availability

The datasets used and/or analyzed during the current study are available from the corresponding author on reasonable request.

## Abbreviations

90a committee: Federal State Committee according to paragraph 90a Social Code Book five
BfArM: Federal Institute for Drugs and Medical Devices
MAUP: Modifiable aria unit problem
ÖGIS: Austrian Health Information System
OSF: Center of Open Science
UPI: Unique Personal Identifier
