# Supplementary material for: Utilization of health-related data in the regional context for health service planning in the Federal State of Brandenburg, Germany—a qualitative study
Source: Res Health Serv Reg. 2024 Sep 25;3:14. doi: 10.1007/s43999-024-00050-0 (PMC11422541; doi:10.1007/s43999-024-00050-0)
Supplement: Supplementary file 1 — Additional file 1: Appendix 1. Standards for Reporting Qualitative Research. Appendix 2. Interview guide. Appendix 3. Translation of selected quotes. [file 43999_2024_50_MOESM1_ESM.pdf]

Appendices

Kugler et al: Utility of health-related data in the regional context for health service planning in the Federal State of Brandenburg, Germany - a qualitative study

Appendix 1: Standards for Reporting Qualitative Research (SRQR) (1)

No	Topic	Item	Indicated on page / line
	**Title and Abstract**		
S1	Title	Concise description of the nature and topic of the study Identifying the study as qualitative or indicating the approach (e.g., ethnography, grounded theory) or data collection methods (e.g., interview, focus group) is recommended	p. 1
S2	Abstract	Summary of key elements of the study using the abstract format of the intended publication; typically includes background, purpose, methods, results, and conclusions	p. 2-3
	**Introduction**		
S3	Problem formulation	Description and significance of the problem/phenomenon studied; review of relevant theory and empirical work; problem statement	p. 4-5
S4	Purpose or research question	Purpose of the study and specific objectives or questions	p. 5
	**Methods**		
S5	Qualitative approach and research paradigm	Qualitative approach (e.g., ethnography, grounded theory, case study, phenomenology, narrative research) and guiding theory if appropriate; identifying the research paradigm (e.g., postpositivist, constructivist/ interpretivist) is also recommended; rationale	Lines 109-110
S6	Researcher characteristics and reflexivity	Researchers' characteristics that may influence the research, including personal attributes, qualifications/experience, relationship with participants, assumptions, and/or presuppositions; potential or actual interaction between researchers' characteristics and the research questions, approach, methods, results, and/or transferability	Lines 111-117
S7	Context	Setting/site and salient contextual factors; rationale	Lines lines 82-90; 119-127
S8	Sampling strategy	How and why research participants, documents, or events were selected; criteria for deciding when no further sampling was necessary (e.g., sampling saturation); rationale	Lines lines 126-132

No	Topic	Item	Indicated on page / line
S9	Ethical issues pertaining to human subjects	Documentation of approval by an appropriate ethics review board and participant consent, or explanation for lack thereof; other confidentiality and data security issues	Lines 171-173
S10	Data collection methods	Types of data collected; details of data collection procedures including (as appropriate) start and stop dates of data collection and analysis, iterative process, triangulation of sources/methods, and modification of procedures in response to evolving study findings; rationale	Lines lines 142-146
S11	Data collections instruments and technologies	Description of instruments (e.g., interview guides, questionnaires) and devices (e.g., audio recorders) used for data collection; if/how the instrument(s) changed over the course of the study	Interview guide: lines 134-137, Table 1, Appendix 2 Devices: lines 143-146
S12	Units of study	Number and relevant characteristics of participants, documents, or events included in the study; level of participation (could be reported in results)	Lines 147-153, table 2
S13	Data processing	Methods for processing data prior to and during analysis, including transcription, data entry, data management and security, verification of data integrity, data coding, and anonymization/deidentification of excerpts	Lines 155-169
S14	Data analysis	Process by which inferences, themes, etc., were identified and developed, including the researchers involved in data analysis; usually references a specific paradigm or approach; rationale	Lines 158-169
S15	Techniques to enhance trustworthiness	Techniques to enhance trustworthiness and credibility of data analysis (e.g., member checking, audit trail, triangulation); rationale	Lines 364-367
	**Results/findings**		
S16	Synthesis and interpretation	Main findings (e.g., interpretations, inferences, and themes); might include development of a theory or model, or integration with prior research or theory	Lines 174-357
S17	Links to empirical data	Evidence (e.g., quotes, field notes, text excerpts, photographs) to substantiate analytic findings	Lines 204-340
	**Discussion**		
S18	Integration with prior work, implications, transferability,	Short summary of main findings; explanation of how findings and conclusions connect to,	Lines 374-433

No	Topic	Item	Indicated on page / line
	and contribution(s) to the field	support, elaborate on, or challenge conclusions of earlier scholarship; discussion of scope of application/ generalizability; identification of unique contribution(s) to scholarship in a discipline or field	
S19	Limitations	Trustworthiness and limitations of findings	Lines 359-373
	**Other**		
S20	Conflicts of interest	Potential sources of influence or perceived influence on study conduct and conclusions; how these were managed	354-356
S21	Funding	Sources of funding and other support; role of funders in data collection, interpretation, and reporting	358

1. O'Brien BC, Harris IB, Beckman TJ, Reed DA, Cook DA. Standards for reporting qualitative research: a synthesis of recommendations. Acad Med. 2014;89(9):1245-51.

Appendix 2: Interview guide

Aspects to check	Specific questions	Maintenance and regulatory issues
**Introduction**		
Presentation<br>Aim of the project<br>Skipping/canceling questions<br>Are there any questions	The aim of the study is to examine which data sources are currently being used for regional health planning in Brandenburg and what challenges exist in the process. In addition, the study aims to capture the needs on the part of health planning and identify the resources that are required.	
START RECORDING		
**Personal data**		
Name<br>Position<br>Organization		
**Block 1: Questions related to health atlases**<br>*Key question*: **Please describe what comes to mind when you hear the term "health atlas"**		
Comprehension question	What do you understand by the term "health atlas"?	
Guiding question: **Could you please describe your experience with health data in a regional context? (e.g. health atlases, databases, series of tables, reports, queries form data sources)**		
What are they used for?<br>Which ones are you aware of	In what context have you already used these data sources?<br>What health atlases or other health-related data with regional relevance do you know (beyond that)?	Could you please give an example?<br>What exactly do you mean by that?<br>Can you describe ... in a little more detail?
**Block 2: Variation in healthcare provision in Brandenburg**<br>*Guiding question*: **If you were to make an assumption: for which areas of health care are there inequalities in Brandenburg?**		
Definition of inequality<br>Issues with healthcare provision in Brandenburg<br>Verify assumption<br>warranted / unwarranted	What does variation mean to you?<br>What specific issues with healthcare provision do you see in Brandenburg?<br><br>How could one verify your assumption [what was said]?<br>How do you distinguish between warranted and unwarranted variation?	Could you provide more information on this?

Aspects to check	Specific questions	Maintenance and regulatory issues
**Block 3: Current data sources in health planning**<br>Guiding question**: Please describe how your organization utilizes data sources for healthcare planning.**		
Typical regional issue<br><br>Which data sources<br><br>External / internal<br><br>Environmental data<br><br><br>Environmental data: utilization<br>Environmental data: data sources<br>Cooperation	Can you name typical questions with regional relevance for your healthcare planning?<br>What data sources does your organization use for healthcare planning?<br><br>Do you also use external / internal data sources (depending on what is mentioned)?<br>To what extent are data on living conditions or environmental data relevant to your healthcare planning? (e.g. heat, air pollution, noise pollution, infrastructure, transportation, traffic, internet coverage)<br>How are these data integrated?<br>Which data sources do you use?<br>To what extent do you cooperate with other organizations in healthcare planning?	Can you tell me more about this?<br>Could you please give an example?<br>What exactly do you mean by that?<br>Can you describe ... in a little more detail?<br>Played ... a role<br>Do you mean ... (to summarize)?
**Block 4: Challenges**<br>*Guiding question*: **Please describe the challenges you face in healthcare planning with regard to available data sources.**		
What <u>is not</u> answered<br><br>What's needed<br><br>Current approach	Which questions can you not answer with the data available to you?<br><br>What data sources would you need to facilitate healthcare planning?<br><br>How are you handling this [the challenges mentioned] at the moment?	

Aspects to check	Specific questions	Maintenance and regulatory issues
**Block 5: Comprehensive health atlas in the future**<br>*Guiding question*: **What do you think of a cross-organizational data source for the regional healthcare planning?**		
Healthcare planning support<br><br>Atlas<br><br>Planned usage	How could a comprehensive data source support your healthcare planning?<br><br>To what extent would the presentation of this data in a health atlas, i.e. as a map, be helpful for your health planning?<br>What would you use such a data source for?	
In case a health atlas endorsed: **How should a comprehensive data source be designed to support your healthcare planning?**		
Type of analysis<br><br>Level of aggregation<br>Indicators<br>Absolute/relative, reference<br>Update interval<br>Environmental data<br><br><br>Variation: measure<br>Variation: warranted/unwarranted	What type of analyses would you like to see? (e.g. mapping of diseases/health / accessibility analyses)<br>How detailed should the data be? (Aggregation levels: counties, districts)<br>Which indicators do you consider relevant?<br>What would be the unit of analysis (absolute/ relative values, reference)?<br>How often should such a data source be updated?<br>Can you elaborate on the extent to which you think environmental data/ data on living conditions should be included in a health atlas for health planning?<br>How can variation be represented in a way that makes sense to you?<br>How could a distinction be made between warranted and unwarranted variation?	Can you tell me more about this?<br>Could you please give an example?<br>What exactly do you mean by that?<br>Can you describe ... in a little more detail?<br>Played ... a role<br>Do you mean ... (to summarize)?

Aspects to check	Specific questions	Maintenance and regulatory issues
In case a health atlas endorsed: **How do you envision the implementation of such a comprehensive data source?**		
Next steps<br>Who is responsible for creation?<br>Publication<br><br>*<u>In case positive for publication</u>*:<br>Target audience<br>Dissemination<br>Media<br><br>Software	How do you envision the next steps towards realization?<br>Which organization should be responsible for the creation?<br>Do you think such a health atlas should be made available to the public?<br><br><br>What ideas do you have regarding the target group?<br>How can the Health Atlas be disseminated?<br>How can the media be involved in the dissemination of the Health Atlas?<br><br>What ideas do you have regarding software?	
In case a health atlas endorsed: **How could a comprehensive data source help to reduce inequalities in health or healthcare?**		
Ensuring action<br><br>Who is responsible for taking action<br><br>Role of the health atlas<br>Example of action	What can be done to ensure that action is taken to minimize inequalities?<br><br>In your opinion, which organization is responsible for taking such actions?<br><br>To what extent could a health atlas contribute to action?<br>Can you give examples of what actions could be taken to reduce inequalities?	Can you tell me more about this?<br>Could you please give an example?<br>What exactly do you mean by that?<br>Can you describe ... in a little more detail?<br>Played ... a role<br>Do you mean ... (to summarize)?
**Block 5: concluding the interview**	**Is there anything else you would like to add to our conversation?**	
**STOP RECORDING**		
Other contacts<br><br>Renewing contact<br>Participation in next steps	Do you have contacts from other healthcare planning organizations that I can contact?<br>Can I contact you again if I have any questions?<br>Would you like to continue participating in the project when it comes to planning further steps together with other organizations?	

Appendix 3: Translation of selected quotes

Subject	German Quote	Translation
Definition health atlas	Es ist im Grunde für mich eine Sammlung von statistischen Daten, Kennzahlen, Indikatoren, die ich eben für meine Arbeit und vor allem für meine Planung nutzen kann. Vor allem natürlich nicht nur in meinem Bezug auf das gesamte Land Brandenburg, sondern natürlich bei uns für die Kommune. Und im besten Fall auch eigentlich noch für den Sozialraum. (T04)	For me, it is basically a collection of statistical data, key figures and indicators that I can use for my work and, in particular, for my planning. Above all, of course, not only in relation to the entire federal state of Brandenburg, but of course for our local authority. And, in the best case, also for the Local neighbourhood. (T04)
Internal data	tendenziell eher unsere eigenen Daten, weil die einfach kleinteilig und näher an uns daran sind, das stimmt schon, und wir gar nicht die Zeit haben, mehr Themen zu bearbeiten. (T10)	generally, rather our own data, because they are simply more detailed and closer to us, that's true, and we don't have the time to address more topics. (T10)
Purpose to use the data: health planning	Wir nutzen jetzt aktuell für das Projekt X, um zielgerichteter unsere Maßnahmen zu steuern. Also, es gab immer so ein bisschen eine Tendenz, (…) [zu] einem Gießkannen-Prinzip. (…) Und die Auswertung unserer Datenanalyse hat gezeigt, dass das ein Fehler ist. Also, dass wir viel größere Betroffenheit, also schlechtere Gesundheitsdaten haben in Berlin-entfernteren Gegenden. (T06)	We are currently using, specifically for Project X, to more effectively control our interventions. So, there has always been a tendency, (…) [towards] a scattergun approach. (…) And the evaluation of our data analysis has shown that this is a mistake. So, we have much greater affection, meaning worse health data, in areas farther away from Berlin. (T06)
Purpose to use the data: public relations and lobbying	Bei der Vorstellung des Mobilitätsatlas. (…) Das ist auch der Presse vorgestellt worden. Und so nutzen wir, ja, in verschiedenen Bereichen publizieren wir, aber nutzen wir auch für Gespräche, auch für Positionspapiere und auch im Rahmen von Veranstaltungen. (T15)	When presenting the Mobility Atlas. (...) This was also presented to the press. And so we use, yes, we publish in various areas, but we also use it for discussions, also for position papers and also as part of events. (T15)
Availability of data	Also da haben wir auf Landesebene das Problem, auf kommunaler Ebene ist das Problem, dass wir gar keine kleinräumigen Daten haben und niemanden haben der sie erheben, auswerten oder verarbeiten könnte. (T07)	So here we have the problem at federal state level, at municipal level the problem is that we have no small-scale data at all and no one who could collect, evaluate or process it. (T07)
Availability of data	Die Angebotsstrukturen der Krankenkassen, das ist für uns	The structures of statutory health insurance providers are sort of black box

	[eine] Blackbox. (…) Wir wissen nicht, an welcher Stelle, welche Krankenkasse ein Gesundheitsförderungsangebot macht. (T06)	for us. (...) We don't know where, which health insurance is offering a health promotion program. (T06)
Awareness of data	ich glaube, wir haben viele Daten. Ich glaube, die Daten sind nicht allen bekannt. (T16)	I think we have a lot of data. I don't think everyone is aware of the data (T16)
Acceptance of data	Mir ist auch aufgefallen, dass es manchmal nicht so einfach ist Entwicklungsdarstellung, Entwicklungsverläufe wirklich für den Zeitraum, den man vielleicht gerne hätte, zu ermitteln, einfach weil sich Definitionen für Kennzahlen, für Indikatoren verändert haben und dann einfach ja, die Vergleichbarkeit nicht mehr gegeben ist. Das hat man schon relativ häufig. Zum Teil ist es ja auch nachvollziehbar, zum Teil setzt man ja selber auch die Definition, schreibt die um, einfach weil es nicht mehr zeitgemäß ist und nicht mehr dem medizinischen State-of-the-Art oder auch dem Public-Health State-of-the-Art entspricht. (T12)	I've also noticed that it's sometimes not so easy to really determine developments and development trends for the period that you would perhaps be interested in, simply because definitions for key figures and indicators have changed and then, quite simply, comparability is no longer given. This happens relatively often. In part, it is also understandable, in part, you set the definition yourself, rewrite it, simply because it is no longer up to date and no longer corresponds to the medical state of the art or the public health state of the art. (T12)
Perceived applicability	Also die Kreise sind für uns zu grob. (…) Brandenburg wurde ja damals so geschnitten, dass viele Brandenburger Landkreise immerhin noch einen Happen ab haben vom Berliner Speckgürtel, ne. (lacht) Und dann aber auch die Peripherie, das ist natürlich jetzt ähm sehr schwierig. Es ist natürlich zu erwarten, dass wir eine starke Heterogenität haben innerhalb eines solchen Landkreises. (T16)	So the districts are too coarse for us. (...) Brandenburg was cut in such a way that many Brandenburg districts still have a slice of the Berlin metropolitan area, right. (laughs) And then there's the periphery, and that's, um, very difficult now, of course. It is of course to be expected that we have a strong heterogeneity within such a district. (T16)
Ability to use	Die Auswertung dieser Daten und da haben wir eine Hülle und Fülle im Gesundheitsamt. Die ließen sich durch die Software, mit der wir arbeiten, statistisch erfassen und auswerten, auch auf Regionalität. Aber wir haben einfach nicht die Zeit uns damit zu befassen. (T03)	The evaluation of this data and we have an enormous amount of it at the local health authority. The software we work with could be used to record and evaluate it statistically, including for regionality. But we simply don't have the time to deal with it. (T03)
Definition of unwanted variation	Ja, die Ungleichheit, ich sage mal, ist auch mit verbunden mit der Erreichbarkeit der	Yes, inequality, I would say, is also connected to the accessibility of healthcare services. The more rural the regions

	Versorgungsangebote, ja. Die ist eben, je ländlicher sie werden, die Regionen, umso weiter müssen einfach die Versicherten auch zu den Angeboten fahren.<br>(T01)	become, the further insured individuals must travel to access the services. (T01)
Definition of unwanted variation	Also sind immer so diese rein rechnerischen Zahlen, die sind da teilweise ein bisschen schwierig - ich sag mal - zu bewerten. Weil nur das reine Angebot heißt ja nicht, dass sie dort auch hinkommen, dass sie einen Termin bekommen, dass sie versorgt werden. (…) aber so eine wirkliche Übersicht liegt niemandem vor. (T13)	So these are always purely numerical figures, and they can be somewhat difficult to assess - I would say. Just because there is a service doesn’t mean they will actually get there, get an appointment, or receive the care they need. (…) but nobody has a real overview. (T13)
Use: Agreeing who should be responsible for action	sagen wir mal so, integrierte Versorgung oder integrierte Planung in der Stadtverwaltung ist leider etwas schwierig<br>(T05)	Let's put it this way, integrated care or integrated planning in city administration is unfortunately somewhat challenging. (T05)
Cross-organizational data source	Das wäre so für uns, bin ich der Meinung, das Nonplusultra, wirklich kleinräumig zu gucken, in der Gemeinde XY, Altersstruktur, Erkrankungsgeschehen, Pflegebedarfe, Pflegediagnosen, das wäre etwas, wo man sagt, in der Gemeinde ist das Thema laut Daten zu fokussieren für entsprechende Projekte. (…) Dass man wirklich die Ressourcen, die Gelder, die irgendwo zur Verfügung stehen, sinnvoll einsetzt. (T08)	In my opinion, that would be the ultimate for us, to really look at a small scale, in the community XY, age structure, incidence of illness, care needs, nursing diagnoses, that would be something where you say, according to the data, the topic is to be focused on in the community for corresponding projects. (...) That you really use the resources, the funds, that are available somewhere, sensibly. (T08)
Cross-organizational data source	Wenn man wirklich eine gemeinsame Zielrichtung hat, indem man diese Daten bewertet, dann finde ich das gut. Aber das haben wir ja nicht, ja, also zumindest aktuell. (T01)	If you really have a common objective by evaluating this data, then I think that's good. But we don't have that, at least not at the moment. (T01)
